# Interference with the Host Haemostatic System by Schistosomes

**DOI:** 10.1371/journal.ppat.1003781

**Published:** 2013-12-26

**Authors:** Mirjam M. Mebius, Perry J. J. van Genderen, Rolf T. Urbanus, Aloysius G. M. Tielens, Philip G. de Groot, Jaap J. van Hellemond

**Affiliations:** 1 Department of Medical Microbiology and Infectious Diseases, Erasmus University Medical Center, Rotterdam, The Netherlands; 2 Department of Internal Medicine and Institute of Tropical Diseases, Harbor Hospital, Rotterdam, The Netherlands; 3 Department of Clinical Chemistry and Haematology, University Medical Center, Utrecht, The Netherlands; International Centre for Genetic Engineering and Biotechnology, India

## Abstract

Schistosomes, parasitic flatworms that cause the tropical disease schistosomiasis, are still a threat. They are responsible for 200 million infections worldwide and an estimated 280,000 deaths annually in sub-Saharan Africa alone. The adult parasites reside as pairs in the mesenteric or perivesicular veins of their human host, where they can survive for up to 30 years. The parasite is a potential activator of blood coagulation according to Virchow's triad, because it is expected to alter blood flow and endothelial function, leading to hypercoagulability. In contrast, hepatosplenic schistosomiasis patients are in a hypocoagulable and hyperfibrinolytic state, indicating that schistosomes interfere with the haemostatic system of their host. In this review, the interactions of schistosomes with primary haemostasis, secondary haemostasis, fibrinolysis, and the vascular tone will be discussed to provide insight into the reduction in coagulation observed in schistosomiasis patients.

Interference with the haemostatic system by pathogens is a common mechanism and has been described for other parasitic worms, bacteria, and fungi as a mechanism to support survival and spread or enhance virulence. Insight into the mechanisms used by schistosomes to interfere with the haemostatic system will provide important insight into the maintenance of the parasitic life cycle within the host. This knowledge may reveal new potential anti-schistosome drug and vaccine targets. In addition, some of the survival mechanisms employed by schistosomes might be used by other pathogens, and therefore, these mechanisms that interfere with host haemostasis might be a broad target for drug development against blood-dwelling pathogens. Also, schistosome antithrombotic or thrombolytic molecules could form potential new drugs in the treatment of haemostatic disorders.

## Introduction

**Figure 1:**
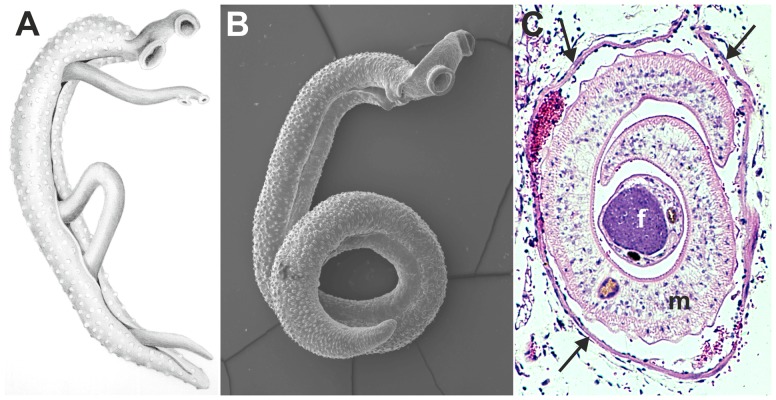
Images of adult schistosomes. Panel A shows a schematic drawing of an adult worm pair. The large adult male embraces the smaller female worm and both worms have two suckers by which they attach to the blood vessel wall. Panel B shows a scanning electron microscope image of a single *S. mansoni* adult male, which is about 1 cm long with a diameter of 1 mm. Panel C shows a cross-section of an adult *S. mansoni* worm pair (m, male; f, female; arrows mark the vessel wall) in a mesenteric venule of a mouse. This cross-section illustrates how close the worm pair is to the vessel wall and suggests the extent to which the worms must disturb blood flow (Panel C is adapted from D. G. Colley and W. E. Secor, PLoS Neglected Tropical Diseases 2007 [10]).

The haemostatic system consists of procoagulant and anticoagulant mechanisms that stop bleeding at sites of blood vessel injury and play an important role in innate immunity [1]–[3]. Procoagulant mechanisms of the haemostatic system can be further divided into primary and secondary haemostasis. Primary haemostasis involves the activation and aggregation of blood platelets, whereas secondary haemostasis involves a cascade of proteolytic reactions that lead to the formation of a stable fibrin clot. Anticoagulant mechanisms of the haemostatic system include inhibitors of primary and secondary haemostasis and the fibrinolytic activity of plasmin that leads to degradation of formed fibrin clots [2]. According to Virchow's triad, three conditions can contribute to the initiation of blood coagulation: normal blood flow is disrupted or altered (stasis); the endothelium is damaged or dysfunctional; and/or the coagulability of blood plasma is increased (hypercoagulability) [4]–[6]. In order to maintain and propagate themselves in blood vessels, many blood-dwelling pathogens not only require adaptations to evade the actions of the host immune system but also need to avoid blood coagulation through interference with the haemostatic system of their host. Schistosomes, blood-dwelling parasitic flatworms, are the cause of the tropical disease schistosomiasis [7]. On average, adult schistosomes reside in their host's bloodstream for three to five years, but their individual lifespan can be as long as 30 years [7]. Schistosomes can be expected to activate coagulation according to Virchow's triad by inducing stasis and alterations in endothelial function [8], [9]. The adult schistosome pair disturbs blood flow due to the large size of the worm pair: 1 cm long with a diameter of 1 mm (Figure 1). Light microscopy images of adult worms inside the mesenteric veins showed that the worm pair occupies the major part of the lumen of the blood vessels in which they reside [8], [10]. This obstruction will induce turbulence in the vein and increase shear stress along the vessel wall. Turbulence has been described to contribute to the formation of thrombi [11]. Furthermore, endothelial cells can be activated by oscillatory blood flow, which is characterized by forward–reverse flow cycles and disrupted blood flow downstream of sites where the vessel lumen is narrowed [12]. This leads to increased expression of molecules involved in blood coagulation and modulation of the vascular tone, such as tissue factor (TF), von Willebrand Factor (VWF), tissue-type plasminogen activator (t-PA), nitric oxide (NO), and prostacyclin (PGI_2_) [13]–[18]. Turbulence and changes in shear stress, induced by the presence of the adult schistosome pair in the blood vessel, could potentially activate platelets and blood coagulation [4], [11]. In addition, although there is no direct evidence of endothelial damage caused by the presence of the adult worm pair in the vein, several studies suggest that schistosomes disturb endothelial cell function, and it has been suggested that the presence of the adult worm in the vein induces endothelial damage [9], [19]–[21]. In murine schistosomiasis, the expression of endothelial NO synthase as well as the production of NO are decreased, which indicates endothelial dysfunction [9], [19], [21]. Furthermore, plasma soluble intercellular adhesion molecule-1 is increased in hepatosplenic schistosomiasis patients, which indicates endothelial activation and inflammation [22]. Extravasation of schistosome eggs may also contribute to endothelial damage or dysfunction, since this disrupts the polarization of the endothelium and causes mobilization and migration of endothelial cells [8]. Therefore, it is likely that parasite-induced alteration in endothelial function or endothelial damage plays a role in activation of blood coagulation. Besides alterations in blood flow and endothelial function, schistosomes have many electronegative charges on their surfaces that could potentially activate platelets and the coagulation cascade, leading to hypercoagulation [23]. Thus, schistosomes have all the characteristics to be potent activators of blood coagulation. However, schistosomiasis patients do not have an increased risk of thrombus formation [24]. In contrast, studies on blood coagulation in hepatosplenic schistosomiasis patients (reviewed by Tanabe [24]) showed that patients have prolonged coagulation times [25]. In infected humans, major haemostatic abnormalities are only observed in hepatosplenic schistosomiasis patients, but murine studies observed changes in the activity of several coagulation factors already during the early phase of schistosomiasis [26]. Hepatosplenic schistosomiasis patients have a reduced activity or reduced levels of the coagulation factors II, VII, IX, X, XI, XII, fibrinogen, high–molecular-weight kininogen (HMWK), and prekallikrein, as well as the regulatory proteins antithrombin and protein C [27], [28]. Furthermore, the levels of thrombin-antithrombin complexes, prothrombin fragment 1+2, plasma fibrinopeptide A, D-dimers, and other fibrin degradation products are increased in these patients [25], [29]. The elevated levels of both markers of coagulation activation (e.g., prothrombin fragment 1+2 and plasma fibrinopeptide A) as well as markers of fibrinolysis (e.g., fibrin degradation products) indicate a continuous activation of both blood coagulation and fibrinolysis in hepatosplenic schistosomiasis patients. Therefore, the observed hypocoagulable and hyperfibrinolytic state of these individuals is the result of both increased consumption of coagulation factors and decreased hepatic synthesis of these factors and cannot solely be attributed to hepatic dysfunction [24], [29]. Also, research showed that blood platelets do not adhere to adult schistosomes or isolated outer surface membranes (tegument) of adult worms [30]. It is thus clear that schistosomes must have mechanisms that suppress the haemostatic response of their host. In this review, the interactions of schistosomes with primary haemostasis, secondary haemostasis, fibrinolysis, and the vascular tone will be discussed in order to provide insight into the reduction in blood coagulation that is observed in schistosomiasis patients.

Identification of schistosome mechanisms that interfere with the haemostatic system provides important insight into the maintenance of the parasitic life cycle within its host. Insight into survival mechanisms of the parasite could provide important clues for novel anti-schistosome drugs or reveal vaccine targets. In addition, other blood-dwelling pathogens face similar survival challenges and may therefore employ similar survival strategies as schistosomes. These mechanisms that interfere with host haemostasis may, therefore, form a broad target for drug development against blood-dwelling pathogens. Also, potent antithrombotic drugs that are currently used in the clinic have been isolated earlier from pathogens, such as streptokinase from *Streptococci* [31]. Schistosome antithrombotic or thrombolytic molecules could therefore form potential novel drugs in the treatment of haemostatic disorders.

## Interference with Primary Haemostasis by Schistosomes

**Figure 2:**
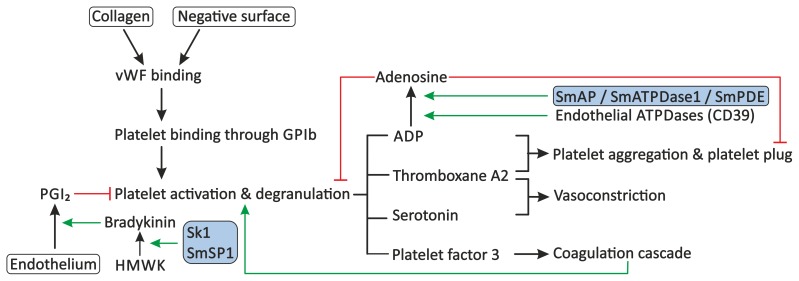
Proposed modulation of primary haemostasis by schistosomes. Primary haemostasis consists of the activation of blood platelets and the formation of a platelet plug. Exposure of subendothelial collagen, due to endothelial damage or the presence of soluble activators or surfaces of pathogens, can facilitate binding of VWF. Platelets adhere to VWF through GPIb present on their surface, followed by their activation and degranulation. Platelets release molecules, such as ADP, thromboxane A2, serotonin, and platelet factor 3, which stimulate further platelet aggregation and activation, stimulate vasoconstriction, and activate secondary haemostasis. Primary haemostasis is inhibited by PGI_2_ from endothelial cells, which inhibits degranulation of platelets, and by the degradation of extracellular ADP to AMP and subsequently to the inhibitor adenosine by endothelial ATPDases, such as CD39. Schistosomes interfere with the primary haemostasis through the proposed degradation of extracellular ADP by SmAP, SmATPDase1, and/or SmPDE, and potentially stimulate the release of PGI_2_ through up-regulation of the production of bradykinin by sK1 or SmSP1. Stimulation is indicated by green arrows. Red lines indicate inhibition. Schistosome proteins are indicated in the shaded boxes. Abbreviations: glycoprotein Ib (GPIb), High-molecular-weight kininogen (HMWK), prostacyclin (PGI_2_), *Schistosoma mansoni* alkaline phosphatase (SmAP), *Schistosoma mansoni* ATP-diphosphohydrolase-1 (SmATPDase1), *Schistosoma mansoni* phosphodiesterase (SmPDE), von Willebrand Factor (VWF).

Primary haemostasis consists of the activation and aggregation of blood platelets. Platelet activation can be triggered by endothelial damage, which leads to exposure of the underlying collagen, or by the presence of soluble activators, such as thrombin or ADP. When the vessel wall is damaged, platelets will adhere to collagen-bound vWF through glycoprotein Ib (GPIb) present on their surface, followed by their activation and degranulation. Under pathophysiological conditions VWF also binds to surfaces of pathogens, such as *Staphylococcus aureus*, and triggers platelet activation [32]. Activated platelets release factors, such as ADP and thromboxane A2, which induce vasoconstriction, stimulate secondary coagulation, and promote further platelet activation and aggregation, resulting in the formation of a stable platelet plug [2]. Several mechanisms have been described that could explain the ability of schistosomes to prevent primary haemostasis (Figure 2).

Ngaiza and Doenhoff observed a decreased platelet count, also called thrombocytopenia, during schistosome infection in mice [33]. This was suggested to contribute to the observed decrease in platelet aggregation around the adult schistosome pair. In schistosomiasis patients, a decreased platelet count was also observed, but symptoms that are commonly observed in patients with thrombocytopenia, e.g., gingival bleeding, are not present in these patients, indicating that platelet aggregation is not fully impaired [34]. Schistosomes must therefore have evolved additional mechanisms to prevent primary haemostasis.

Extracellular ADP induces platelet aggregation. ADP-mediated platelet aggregation is normally controlled by ATP-diphosphohydrolase (ATPDase) proteins, such as CD39/ATPDase1, that are present on endothelial cells [35], [36]. These ATPDases hydrolyse ATP to ADP and ADP to AMP and subsequently to adenosine. This degradation of ADP and the subsequent formation of the inhibitor adenosine leads to inhibition of ADP-mediated platelet activation and aggregation [37], [38]. The schistosome tegument contains several enzymatic activities that could lead to the degradation of extracellular ATP or ADP [39]. Alkaline phosphatase activity is present in the tegument of *Schistosoma mansoni*, and recombinant *S. mansoni* alkaline phosphatase (SmAP) has been characterized [40], [41]. Alkaline phosphatase enzymes are present in many organisms and hydrolyze a broad spectrum of substrates, including ATP, ADP, and AMP [42]. *S. mansoni* alkaline phosphatase (SmAP) has structural homology to human placental alkaline phosphatase, which suggests that its substrate specificity is similar to human alkaline phosphatases. In addition, investigation of the *S. mansoni* tegument revealed phosphodiesterase (SmPDE) activity, which hydrolyzes, among others, ATP and AMP [43]. Furthermore, a tegument-localized *S. mansoni* ATP-diphosphohydrolase-1 (SmATPDase1) activity was characterized, which is capable of hydrolysis of both ATP and ADP to AMP [44], [45]. Therefore, these tegumental enzymes could, by mimicking human ATPDases, form a potential strategy to inhibit platelet activation.

Furthermore, the actions of an *S. mansoni* protein with kallikrein-like activity, sK1, and a protein with homology to mouse plasma kallikrein, SmSP1, may form a mechanism through which schistosomes inhibit primary haemostasis [46], [47]. Kallikrein is able to convert HMWK to the small vasoactive peptide bradykinin, which is a potent vasodilator [20], [48]. Bradykinin can trigger the release of prostacyclin (PGI_2_) from endothelial cells [48]. PGI_2_ is another potent vasodilator but also inhibits platelet degranulation. Exposure on the surface or secretion of the tegumental proteins sK1 and SmSP1 has been suggested, and it has been shown that sK1 is able to convert HMWK to bradykinin in vitro, which suggests the production of bradykinin by these proteins also during schistosome infection in vivo [46], [47]. Kallikrein-like activity has not been described for SmSP1, but its homology to mouse kallikrein suggests kallikrein-like activity. sK1, and potentially SmSP1, could thus play a role in the inhibition of primary haemostasis by the parasite, although such a role has not been demonstrated.

## Interference with Secondary Haemostasis by Schistosomes

**Figure 3:**
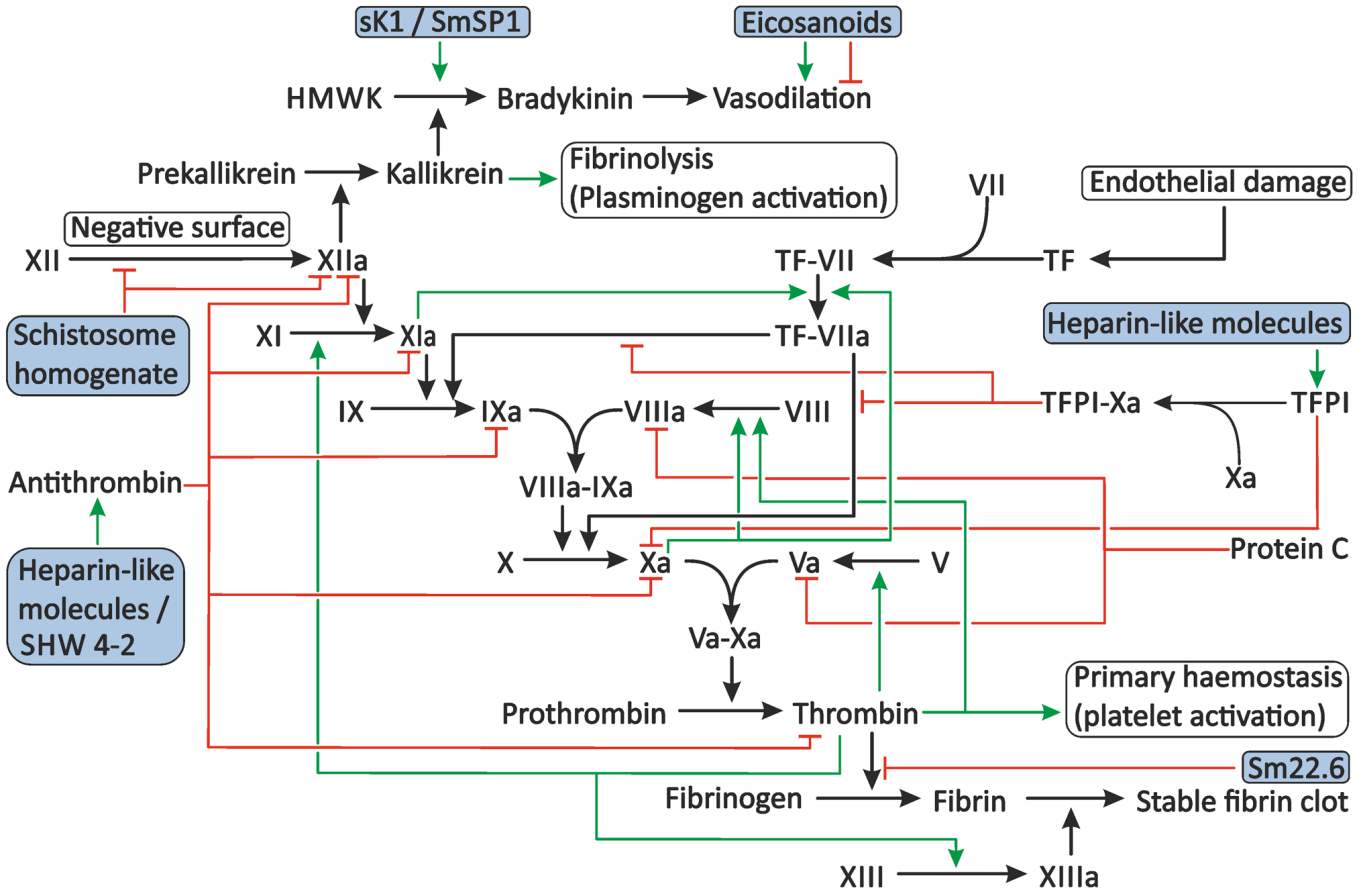
Proposed modulation of secondary haemostasis and vascular tone by schistosomes. Secondary haemostasis can be activated through two different pathways: either by the presence of TF or by contact of coagulation factors with collagen, pathogens, or other negatively charged surfaces. This triggers a cascade of cleavage reactions, ultimately leading to the cleavage of fibrinogen to fibrin and the formation of a stable fibrin clot. This process is regulated by antithrombin, TFPI, and protein C. Schistosomes potentially interfere with secondary haemostasis at several steps in the cascade. Schistosome whole worm homogenate blocks the conversion of XII to XIIa and inhibits the actions of XIIa. The proteolytic activity of thrombin is inhibited by the schistosome antigen Sm22.6. Furthermore, schistosome heparin-like glycosaminoglycans may enhance the activity of antithrombin and, possibly, TFPI, and the schistosome serpin SHW 4-2 might mimic human antithrombin. The vascular tone can be influenced by schistosomes through the production of both vasodilating and vasoconstricting eicosanoids and the presence of sK1 and SmSP1 that could potentially convert HMWK into the vasodilator bradykinin. The green arrows indicate stimulation. Inhibition is indicated by the red lines. The shaded boxes indicate schistosome proteins. Abbreviations: activated coagulation factor XII (XIIa), coagulation factor XII (XII), High-molecular-weight kininogen (HMWK), tissue factor (TF), tissue factor pathway inhibitor (TFPI).

Secondary haemostasis consists of two pathways: the tissue factor (TF) pathway (extrinsic pathway) and the contact activation (intrinsic) pathway [2], [49]. The extrinsic pathway is considered to be the physiologically most relevant pathway and is induced by the exposure of TF after endothelial damage, but under pathophysiological conditions it can also be activated by TF expressed on immune cells or endothelial cells [3], [50]. The intrinsic pathway is activated by binding of coagulation factor XII (XII) to collagen or negatively charged surfaces [51], [52]. Local accumulation of XII leads to its auto-activation and induces a cascade of cleavage reactions that activate other coagulation factors [53]. Finally, both the extrinsic and intrinsic pathways lead to the cleavage of fibrinogen to fibrin by thrombin and the formation of a stable fibrin clot (Figure 3).

## Interference with the Activation of Secondary Haemostasis by Schistosomes

Schistosomes are potential activators of the extrinsic pathway due to the induction of turbulence and endothelial damage and/or alteration of endothelial function induced by the adult parasite and/or the eggs [8], [9], [16]. Furthermore, elevated levels of tumor necrosis factor alpha (TNF-α) are present in schistosomiasis patients, and high TNF-α levels are known to induce TF expression on endothelial cells in vitro [50], [54]. In addition, increased TF expression is present in monocytes from hepatosplenic schistosomiasis patients, compared to monocytes of healthy donors, suggesting the involvement of monocyte TF expression in the prothrombotic state observed in schistosomiasis patients [54]. However, to date there is no evidence that schistosomes interfere with extrinsic coagulation [55].

On the other hand, in vitro experiments show that schistosomes can inhibit the activation of secondary haemostasis in at least two steps: the activity of thrombin and the activation and activity of XII, a factor of the intrinsic pathway, suggesting inhibition of secondary haemostasis during schistosome infection in vivo [55]–[58]. Both the extrinsic and the intrinsic pathway lead to the activation of thrombin. The inhibition of thrombin activity by schistosomes could therefore be a potential strategy to prevent the formation of fibrin clots. The *S. mansoni* antigen Sm22.6 discovered by Stein and David is expressed in the cytoplasmic layer of the schistosome tegument but is also present in the host circulation [59]. The exact role of Sm22.6 in the parasitic life cycle is unknown, but the protein interacts with thrombin and inhibits its protease activity [58]. This suggests that Sm22.6 could prevent the formation of fibrin clots around the adult parasite [58]. Furthermore, thrombin plays an important role in the amplification loop of the coagulation cascade through the activation of XI by thrombin; blocking this protein could interfere with this positive feedback mechanism.

Schistosomes have also been observed to interfere with the intrinsic pathway. Whole worm homogenate of *S. mansoni* inhibits both the activation and activity of XIIa [55]–[57]. The molecules responsible for this inhibitory activity have not been characterized, and their localization in the adult worm is, therefore, not known. Further investigations are required to confirm this suggested activity. Furthermore, the importance of XII in coagulation in vivo is still debated [60]. XII deficiency does not result in a bleeding tendency in vivo, in contrast to a decreased coagulation time observed in in vitro tests [61]. The reason for this discrepancy remains unclear, but it led to the assumption that XII is not required for normal haemostasis, although a role has been suggested in pathological thrombus formation [60]. It should therefore be stressed that the role of inhibition of XII in prevention of coagulation during schistosome infections might be very limited. Another possibility could be that inhibition of XII by schistosomes is not involved in interference with haemostasis, but instead functions in the evasion of immune responses by the parasite. XII is involved in the activation of the complement cascade, and inhibition of XII by schistosomes might thus be an example of an immune-evasion strategy rather than a strategy to prevent blood coagulation [62].

## Interference with Regulatory Mechanisms of Secondary Haemostasis by Schistosomes

Secondary coagulation is regulated by the actions of three proteins: antithrombin, tissue factor pathway inhibitor (TFPI), and protein C. Stimulation or mimicking of host mechanisms for the regulation of blood coagulation could form a potential strategy employed by schistosomes to interfere with haemostasis.

The serine protease inhibitor (serpin) antithrombin is the major inhibitor of coagulation proteases [61]. Antithrombin is constitutively present in blood plasma and binds and inactivates thrombin, as well as coagulation factors IXa, Xa, and XIa. The efficiency of thrombin binding increases 2,000- to 10,000-fold when antithrombin associates with its cofactor heparin [61]. Schistosomes could potentially increase antithrombin activity by expressing heparin or heparin-like molecules, such as heparan sulfate. Heparan sulfate is a heparin-like molecule that is present on the surface of endothelial cells and is responsible for the anticoagulant properties of the endothelium. Heparin-like glycosaminoglycans, i.e., heparan sulfate and dermatan sulfate, are present in tegument fractions of *S. mansoni* [63]. However, the localization of these heparin-like molecules on the outer tegumental membrane and the capacity of these molecules to interfere with the haemostatic system have not been determined.

Furthermore, Blanton et al. identified a serpin in *S. haematobium*, SHW 4-2, with high sequence similarity to antithrombin and glial-derived nexins, which can both bind and inhibit thrombin [64], [65]. Surface localization of SHW 4-2 was shown by immunolocalization, suggesting that *S. haematobium* serpin may mimic the actions of antithrombin and could play a role in the inhibition of secondary haemostasis.

TFPI, the natural regulator of TF activity, is present in blood plasma and platelets, and is synthesized by endothelial cells. Its mode of action is peculiar; TFPI binds and inhibits coagulation factor Xa. This TFPI-Xa complex subsequently binds to the TF-VIIa complex, thereby inhibiting further activity of coagulation factor VIIa. TFPI present in plasma is active, but present in low concentrations and only able to delay the coagulation cascade. TFPI can be released by degranulation of activated platelets and/or from the endothelium by heparin and thereby regulate TF activity [2], [66]. The presence of heparin-like molecules on the outer surface of adult schistosomes suggests that the parasite may stimulate the release and local accumulation of TFPI from the endothelium, although this has not been confirmed.

## Stimulation of Fibrinolysis by Schistosomes

**Figure 4:**
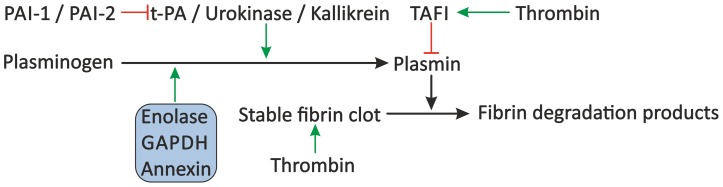
Proposed modulation of the fibrinolytic system by schistosomes. The fibrinolytic system inhibits blood coagulation through degradation of the formed fibrin clot by plasmin. Plasminogen is cleaved to plasmin by plasma kallikrein, but mainly by t-PA and urokinase, which are inhibited by PAI-1 and PAI-2. Furthermore, plasmin activity is inhibited by TAFI. Schistosomes may stimulate fibrinolysis by the presence of enolase, GAPDH, and annexin, which bind plasminogen and facilitate its conversion to plasmin by t-PA. Green arrows indicate stimulation. Inhibition is indicated by the red lines. Schistosome proteins are denoted in the shaded box. Abbreviations: tissue-type plasminogen activator (t-PA), plasminogen activator inhibitor 1 (PAI-1) and 2 (PAI-2), thrombin-activatable fibrinolysis inhibitor (TAFI), glyceraldehyde-3-phosphate dehydrogenase (GAPDH).

Besides interference with primary or secondary haemostasis, schistosomes may also reduce thrombus formation through stimulation of fibrinolytic pathways. Fibrinolysis by plasmin controls the degradation of fibrin clots to fibrin degradation products (Figure 4). The proteolytic activation of plasminogen to plasmin is stimulated by plasma kallikrein, but mainly by urokinase and t-PA, which are slowly released from damaged endothelium [2], [67]. Although several reports show no enhancement of fibrinolysis by *S. mansoni* [55], [56], more recent reports indicate the presence of activators of plasminogen at the surface of *S. bovis* [68]–[70]. Ramajo-Hernández et al. screened tegument fractions for proteins binding plasminogen and demonstrated that tegument fractions enhanced the generation of plasmin by t-PA [68]. Ten proteins that were able to bind plasminogen were identified; the most prominent ones were enolase, glyceraldehyde-3-phosphate dehydrogenase (GAPDH), and actin. Surface localization was shown for GAPDH and enolase but not for actin [69], [71]. Enolase is expressed on the surface of male schistosomes only, and recombinantly expressed enolase has been shown to bind plasminogen and enhance its conversion to plasmin in the presence of t-PA [68], [69]. Similarly, Yang et al. discovered a plasminogen-binding enolase in *S. japonicum*, indicating that plasminogen-binding by schistosome enolases may be a common feature of schistosomes [72]. The role of GAPDH and actin in the conversion of plasminogen is still unclear, but it has been described that interaction of plasminogen with other molecules induces conformational changes in plasminogen which could aid in the conversion of plasminogen to plasmin by t-PA [73]. Further research by de la Torre-Escudero et al. revealed on the parasitic surface the presence of a schistosome protein belonging to the family of annexins [70]. Annexins have many functions, and some annexins, such as human annexin-A2, are involved in the regulation of fibrinolysis [74]. Schistosome annexin binds plasminogen and enhances the t-PA mediated conversion of plasminogen to plasmin [70]. The schistosome surface annexin may thus have a role in local activation of fibrinolysis during schistosome infection, thereby stimulating the degradation of thrombin clots that could be formed at the parasite's surface.

## Interference with the Vascular Tone by Schistosomes

Besides a possible activation of blood coagulation by the parasite, the parasite faces a second problem in the bloodstream. The eggs deposited by the female are laid in the small veins surrounding the intestine, and the parasite pair is known to wander between the large portal vein and these small veins [75]. This migration could, due to the large size of the adult worm pair compared to the size of the blood vessels, temporarily cause obstruction of blood flow, and the resulting changes in blood flow could potentially damage the endothelium and surrounding tissues. Manipulation of the vascular tone, e.g., by inducing vasodilation, could prevent damage caused by obstruction of these small blood vessels, and such a strategy would therefore be advantageous for the parasite. Several mechanisms have been proposed by which schistosomes could modulate vascular tone (Figure 3) (reviewed in detail by Da'dara and Skelly [20]). For example, schistosomes are able to produce and secrete eicosanoids. Eicosanoids have diverse functions; among these is influencing the vascular tone. *S. mansoni* produces eicosanoids that stimulate vasodilation (e.g., the cyclo-oxygenase products prostaglandin D_2_ and prostaglandin E_2_), as well as 5-lipoxygenase products that are able to induce vasoconstriction [e.g., leukotriene C_4_ and 15-hydroxyeicosatetraenoic acid (15-HETE)] [76]. It has been shown that schistosome vasoconstrictors are released in smaller amounts than the vasodilators [76], [77]. This suggests that eicosanoids play a role in the stimulation of vasodilation during infection. However, this difference in production of vasoconstriction-promoting and vasodilation-promoting eicosanoids could also merely reflect differences in biological activity, and therefore, the true impact of schistosome eicosanoids on the vascular tone during infection remains unclear.

Another potential mechanism to induce vasodilation by schistosomes is through the actions of the schistosome proteins sK1 and SmSP1 [46], [47]. sK1 is a schistosome protein with kallikrein-like activity, and SmSP1 has homology to mouse kallikrein, but kallikrein-like activity has not been described for SmSP1 [46], [47]. As discussed before, *S. mansoni* sK1 and, potentially, SmSP1 convert kininogen to the potent vasodilator bradykinin [46].

## Concluding Remarks

According to Virchow's triad, schistosomes are expected to be potent activators of blood coagulation. However, the parasite has evolved several mechanisms to actively inhibit blood coagulation and manipulate the vascular tone, ensuring long-term survival in the host's bloodstream (Table 1). Many experiments showed that primary and secondary haemostasis can be inhibited by the parasite through a combination of schistosome-specific mechanisms that inhibit key steps in the coagulation cascade and by schistosome proteins that exploit host mechanisms for regulation of haemostasis. Furthermore, schistosome proteins may actively stimulate fibrinolysis and manipulate vascular tone. The many strategies used by schistosomes to interfere with the haemostatic system of their host reflect the complex host–parasite relationship. Although some major discoveries in manipulation of blood coagulation by schistosomes have been made, additional studies are required to further understand these mechanisms and determine their importance during infection. This would provide more insight into pathways involved in maintaining the parasitic life cycle within the host and may reveal new targets for the development of anti-schistosome drugs or vaccines. Furthermore, mechanisms described here might also be applicable to other blood-dwelling pathogens and may reveal broad targets for the development of drugs to be used against these pathogens. In addition, schistosome molecules that interfere with the haemostatic system could form potential new antithrombotic or thrombolytic drugs for the treatment of haemostatic disorders.

**Table 1:** Proposed interference with the haemostatic system and vascular tone by schistosome molecules.

Target	Schistosome molecule	Effect
*Hypocoagulation mechanisms*		
**Primary haemostasis**		
ADP	SmAP [40], [41]	Hydrolysis of ADP to AMP and adenosine, a competitive inhibitor of platelet aggregation and degranulation.
	SmATPDase1 [44], [45]	Hydrolysis of ATP and ADP to AMP.
	SmPDE [43]	Hydrolysis of ATP and AMP.
HMWK	sK1 [46]	Conversion of HMWK to bradykinin. Bradykinin stimulates the release of PGI_2_, an inhibitor of platelet degranulation, from endothelial cells.
	SmSP1 [47]	Homology to mouse plasma kallikrein.
**Secondary haemostasis**		
Factor XII	Schistosome homogenate [55]–[57]	Inhibition of the conversion of coagulation factor XII to XIIa and the actions of XIIa.
Thrombin	Sm22.6 [58]	Inhibitor of thrombin activity.
Antithrombin	Heparin-like molecules [63]	Potential increase of the activity of antithrombin.
	SHW 4-2 [65]	Sequence similarity to antithrombin.
*Hyperfibrinolytic mechanisms*		
Plasminogen	Enolase [68], [69], [72]	Binding and conversion of plasminogen to plasmin by t-PA.
	GAPDH [68]	Binding of plasminogen.
	Annexin [70]	Binding and conversion of plasminogen to plasmin by t-PA.
*Vascular tone*		
HMWK	sK1 [46]	Conversion of HMWK to bradykinin.
	SmSP1 [47]	Homology to mouse plasma kallikrein.
Vasodilation	Eicosanoids [20]	Both vasodilating and vasoconstricting effects.

## References

[1] Klinger MH, Jelkmann W (2002) Role of blood platelets in infection and inflammation. J Interferon Cytokine Res 22: 913–922. 12396713 10.1089/10799900260286623

[2] Hoffbrand AV, Pettit JE, Moss PAH (2003) Essential haematology. Hoboken: Blackwell Science. 349 p.

[3] Opal SM (2003) Interactions between coagulation and inflammation. Scand J Infect Dis 35: 545–554. 14620133 10.1080/00365540310015638

[4] Lowe GD (2003) Virchow's triad revisited: Abnormal flow. Pathophysiol Haemost Thromb 33: 455–457. 15692260 10.1159/000083845

[5] Bagot CN, Arya R (2008) Virchow and his triad: A question of attribution. Br J Haematol 143: 180–190. 18783400 10.1111/j.1365-2141.2008.07323.x

[6] Wolberg AS, Aleman MM, Leiderman K, Machlus KR (2012) Procoagulant activity in hemostasis and thrombosis: Virchow's triad revisited. Anesth Analg 114: 275–285. 22104070 10.1213/ANE.0b013e31823a088c PMC3264782

[7] Gryseels B, Polman K, Clerinx J, Kestens L (2006) Human schistosomiasis. Lancet 368: 1106–1118. 16997665 10.1016/S0140-6736(06)69440-3

[8] File S (1995) Interaction of schistosome eggs with vascular endothelium. J Parasitol 81: 234–238. 7707199

[9] Silva CL, Morel N, Noel F (1998) Portal veins of mice infected with *Schistosoma mansoni* exhibit an increased reactivity to 5-hydroxytryptamine. Mem Inst Oswaldo Cruz 93 (Suppl 1) 153–155. 9921337 10.1590/s0074-02761998000700021

[10] Colley DG, Secor WE (2007) A schistosomiasis research agenda. PLoS Negl Trop Dis 1: e32 doi: 10.1371/journal.pntd.0000032 18060081 10.1371/journal.pntd.0000032 PMC2154383

[11] Stein PD, Sabbah HN (1974) Measured turbulence and its effect on thrombus formation. Circ Res 35: 608–614. 4278187 10.1161/01.res.35.4.608

[12] Johnson BD, Mather KJ, Wallace JP (2011) Mechanotransduction of shear in the endothelium: Basic studies and clinical implications. Vasc Med 16: 365–377. 22003002 10.1177/1358863X11422109

[13] Diamond SL, Eskin SG, McIntire LV (1989) Fluid flow stimulates tissue plasminogen activator secretion by cultured human endothelial cells. Science 243: 1483–1485. 2467379 10.1126/science.2467379

[14] Galbusera M, Zoja C, Donadelli R, Paris S, Morigi M, et al (1997) Fluid shear stress modulates von willebrand factor release from human vascular endothelium. Blood 90: 1558–1564. 9269774

[15] Sun RJ, Muller S, Wang X, Zhuang FY, Stoltz JF (2000) Regulation of von willebrand factor of human endothelial cells exposed to laminar flows: An *in vitro* study. Clin Hemorheol Microcirc 23: 1–11. 11214708

[16] Mazzolai L, Silacci P, Bouzourene K, Daniel F, Brunner H, et al (2002) Tissue factor activity is upregulated in human endothelial cells exposed to oscillatory shear stress. Thromb Haemost 87: 1062–1068. 12083487

[17] Jin ZG, Ueba H, Tanimoto T, Lungu AO, Frame MD, et al (2003) Ligand-independent activation of vascular endothelial growth factor receptor 2 by fluid shear stress regulates activation of endothelial nitric oxide synthase. Circ Res 93: 354–363. 12893742 10.1161/01.RES.0000089257.94002.96

[18] Walshe TE, Ferguson G, Connell P, O'Brien C, Cahill PA (2005) Pulsatile flow increases the expression of eNOS, ET-1, and prostacyclin in a novel *in vitro* coculture model of the retinal vasculature. Invest Ophthalmol Vis Sci 46: 375–382. 15623798 10.1167/iovs.04-0806

[19] Silva CL, Lenzi HL, Silva VF, Paulo FO, Noel F (2003) Cellular mechanisms involved in the increased contraction of portal veins from *Schistosoma mansoni*-infected mice. Parasitol Res 89: 16–22. 12474038 10.1007/s00436-002-0711-7

[20] Da'dara A, Skelly PJ (2011) Manipulation of vascular function by blood flukes? Blood Rev 25: 175–179. 21543145 10.1016/j.blre.2011.04.002 PMC3113519

[21] Oliveira SD, Quintas LE, Amaral LS, Noel F, Farsky SH, et al (2011) Increased endothelial cell-leukocyte interaction in murine schistosomiasis: Possible priming of endothelial cells by the disease. PLoS One 6: e23547 doi: 10.1371/journal.pone.0023547 21853150 10.1371/journal.pone.0023547 PMC3154496

[22] Esterre P, Raobelison A, Ramarokoto CE, Ravaoalimalala VE, Boisier P, et al (1998) Serum concentrations of sICAM-1, sE-, sP- and sL-selectins in patients with *Schistosoma mansoni* infection and association with disease severity. Parasite Immunol 20: 369–376. 9767602 10.1046/j.1365-3024.1998.00168.x

[23] Stein PC, Lumsden RD (1973) *Schistosoma mansoni*: Topochemical features of cercariae, schistosomula, and adults. Exp Parasitol 33: 499–514. 4123959 10.1016/0014-4894(73)90118-5

[24] Tanabe M (2003) Haemostatic abnormalities in hepatosplenic schistosomiasis mansoni. Parasitol Int 52: 351–359. 14665393 10.1016/s1383-5769(03)00051-5

[25] Omran SA, el-Bassiouni NE, Hussein NA, Akl MM, Hussein AT, et al (1995) Disseminated intravascular coagulation in endemic hepatosplenic schistosomiasis. Haemostasis 25: 218–228. 7489960 10.1159/000217164

[26] Carvalho MG, Mello RT, Soares AL, Bicalho RS, Lima e Silva FC, et al (2005) Murine schistosomiasis mansoni: Process of blood coagulation at pre-patent, acute and chronic phases, and consequence of chemotherapeutic cure on the reversion of changes. Blood Coagul Fibrinolysis 16: 469–475. 16175005 10.1097/01.mbc.0000179911.73032.d5

[27] Amin HM, Omran SA, el-Bassuoni NE, el-Kaliouby AH, el-Ashmawy SA (1994) Assessment of factors II, VII, IX, X, and protein C in hepatosplenic schistosomiasis. Haemostasis 24: 22–26. 7959352 10.1159/000217076

[28] El-Bassiouni NE, El Bassiouny AE, Hussein NA, El-Sayed HH, Ibrahim IM, et al (1998) The coagulation profile in hepatosplenic schistosomiasis. Blood Coagul Fibrinolysis 9: 189–194. 9622218 10.1097/00001721-199803000-00011

[29] El-Bassiouni NE, el Bassiouny AE, el-Khayat HR, Akl MM, Omran SA (1996) Hyperfibrinolysis in hepatosplenic schistosomiasis. J Clin Pathol 49: 990–993. 9038736 10.1136/jcp.49.12.990 PMC499647

[30] Wu YP, Lenting PJ, Tielens AGM, de Groot PG, van Hellemond JJ (2007) Differential platelet adhesion to distinct life-cycle stages of the parasitic helminth *Schistosoma mansoni* . J Thromb Haemost 5: 2146–2148. 17883706 10.1111/j.1538-7836.2007.02725.x

[31] Sun H (2006) The interaction between pathogens and the host coagulation system. Physiology (Bethesda) 21: 281–288. 16868317 10.1152/physiol.00059.2005

[32] Herrmann M, Hartleib J, Kehrel B, Montgomery RR, Sixma JJ, et al (1997) Interaction of von willebrand factor with *Staphylococcus aureus* . J Infect Dis 176: 984–991. 9333157 10.1086/516502

[33] Ngaiza JR, Doenhoff MJ (1987) *Schistosoma mansoni*-induced thrombocytopenia in mice. Trans R Soc Trop Med Hyg 81: 655–656. 3127967 10.1016/0035-9203(87)90444-5

[34] Correia MC, Domingues AL, Lacerda HR, Santos EM, Machado CG, et al (2009) Platelet function and the von willebrand factor antigen in the hepatosplenic form of schistosomiasis mansoni. Trans R Soc Trop Med Hyg 103: 1053–1058. 19118853 10.1016/j.trstmh.2008.11.017

[35] Kaczmarek E, Koziak K, Sevigny J, Siegel JB, Anrather J, et al (1996) Identification and characterization of CD39/vascular ATP diphosphohydrolase. J Biol Chem 271: 33116–33122. 8955160 10.1074/jbc.271.51.33116

[36] Sevigny J, Sundberg C, Braun N, Guckelberger O, Csizmadia E, et al (2002) Differential catalytic properties and vascular topography of murine nucleoside triphosphate diphosphohydrolase 1 (NTPDase1) and NTPDase2 have implications for thromboregulation. Blood 99: 2801–2809. 11929769 10.1182/blood.v99.8.2801

[37] Born GV (1962) Aggregation of blood platelets by adenosine diphosphate and its reversal. Nature 194: 927–929. 13871375 10.1038/194927b0

[38] Born GV, Cross MJ (1963) The aggregation of blood platelets. J Physiol 168: 178–195. 14056485 10.1113/jphysiol.1963.sp007185 PMC1359417

[39] Bhardwaj R, Skelly PJ (2009) Purinergic signaling and immune modulation at the schistosome surface? Trends Parasitol 25: 256–260. 19423396 10.1016/j.pt.2009.03.004

[40] Cesari IM, Simpson AJ, Evans WH (1981) Properties of a series of tegumental membrane-bound phosphohydrolase activities of *Schistosoma mansoni* . Biochem J 198: 467–473. 6275849 10.1042/bj1980467 PMC1163290

[41] Araujo-Montoya BO, Rofatto HK, Tararam CA, Farias LP, Oliveira KC, et al (2011) *Schistosoma mansoni*: Molecular characterization of alkaline phosphatase and expression patterns across life cycle stages. Exp Parasitol 129: 284–291. 21784070 10.1016/j.exppara.2011.07.008

[42] Millan JL (2006) Alkaline phosphatases: Structure, substrate specificity and functional relatedness to other members of a large superfamily of enzymes. Purinergic Signal 2: 335–341. 18404473 10.1007/s11302-005-5435-6 PMC2254479

[43] Bogitsh BJ, Krupa PL (1971) *Schistosoma mansoni* and *Haematoloechus medioplexus*: Nuclosidediphosphatase localization in tegument. Exp Parasitol 30: 418–425. 4333150 10.1016/0014-4894(71)90106-8

[44] Vasconcelos EG, Nascimento PS, Meirelles MN, Verjovski-Almeida S, Ferreira ST (1993) Characterization and localization of an ATP-diphosphohydrolase on the external surface of the tegument of *Schistosoma mansoni* . Mol Biochem Parasitol 58: 205–214. 8479445 10.1016/0166-6851(93)90042-v

[45] DeMarco R, Kowaltowski AT, Mortara RA, Verjovski-Almeida S (2003) Molecular characterization and immunolocalization of *Schistosoma mansoni* ATP-diphosphohydrolase. Biochem Biophys Res Commun 307: 831–838. 12878186 10.1016/s0006-291x(03)01268-3

[46] Carvalho WS, Lopes CT, Juliano L, Coelho PM, Cunha-Melo JR, et al (1998) Purification and partial characterization of kininogenase activity from *Schistosoma mansoni* adult worms. Parasitology 117: 311–319. 9820852 10.1017/s0031182098003175

[47] Cocude C, Pierrot C, Cetre C, Fontaine J, Godin C, et al (1999) Identification of a developmentally regulated *Schistosoma mansoni* serine protease homologous to mouse plasma kallikrein and human factor I. Parasitology 118: 389–396. 10340330 10.1017/s0031182098003874

[48] Maurer M, Bader M, Bas M, Bossi F, Cicardi M, et al (2011) New topics in bradykinin research. Allergy 66: 1397–1406. 21859431 10.1111/j.1398-9995.2011.02686.x

[49] Norris LA (2003) Blood coagulation. Best Pract Res Clin Obstet Gynaecol 17: 369–383. 12787532 10.1016/s1521-6934(03)00014-2

[50] Nawroth PP, Stern DM (1986) Modulation of endothelial cell hemostatic properties by tumor necrosis factor. J Exp Med 163: 740–745. 3753996 10.1084/jem.163.3.740 PMC2188058

[51] Wilner GD, Nossel HL, LeRoy EC (1968) Activation of hageman factor by collagen. J Clin Invest 47: 2608–2615. 4302176 10.1172/JCI105943 PMC297431

[52] Espana F, Ratnoff OD (1983) Activation of hageman factor (factor XII) by sulfatides and other agents in the absence of plasma proteases. J Lab Clin Med 102: 31–45. 6343536

[53] Renne T, Schmaier AH, Nickel KF, Blomback M, Maas C (2012) *In vivo* roles of factor XII. Blood 120: 4296–4303. 22993391 10.1182/blood-2012-07-292094 PMC3507141

[54] Amer A, Amer ME (2002) Enhanced monocyte tissue factor expression in hepatosplenic schistosomiasis. Blood Coagul Fibrinolysis 13: 43–47. 11994566 10.1097/00001721-200201000-00006

[55] Tsang VC, Hubbard WJ, Damian RT (1977) Coagulation factor XIIa (activated hageman factor) inhibitor from adult *Schistosoma mansoni* . Am J Trop Med Hyg 26: 243–247. 848647 10.4269/ajtmh.1977.26.243

[56] Tsang VC, Damian RT (1977) Demonstration and mode of action of an inhibitor for activated hageman factor (factor XIIa) of the intrinsic blood coagulation pathway from *Schistosoma mansoni* . Blood 49: 619–633. 843620

[57] Foster CB, Flanigan TP, DeStigter KK, Blanton R, Dumenco LL, et al (1992) Inhibition of the activation of hageman factor (factor XII) by extracts of *Schistosoma mansoni* . J Lab Clin Med 120: 735–739. 1431502

[58] Lin YL, He S (2006) Sm22.6 antigen is an inhibitor to human thrombin. Mol Biochem Parasitol 147: 95–100. 16499980 10.1016/j.molbiopara.2006.01.012

[59] Stein LD, David JR (1986) Cloning of a developmentally regulated tegument antigen of *Schistosoma mansoni* . Mol Biochem Parasitol 20: 253–264. 2429181 10.1016/0166-6851(86)90106-4

[60] Renne T, Pozgajova M, Gruner S, Schuh K, Pauer HU, et al (2005) Defective thrombus formation in mice lacking coagulation factor XII. J Exp Med 202: 271–281. 16009717 10.1084/jem.20050664 PMC2213000

[61] Beck WS (1985) Hematology. Cambridge: The MIT Press. 496 p.

[62] Ghebrehiwet B, Silverberg M, Kaplan AP (1981) Activation of the classical pathway of complement by hageman factor fragment. J Exp Med 153: 665–676. 7252410 10.1084/jem.153.3.665 PMC2186101

[63] Robertson NP, Cain GD (1985) Isolation and characterization of glycosaminoglycans from *Schistosoma mansoni* . Comp Biochem Physiol B 82: 299–306. 4053588 10.1016/0305-0491(85)90245-7

[64] Evans DL, McGrogan M, Scott RW, Carrell RW (1991) Protease specificity and heparin binding and activation of recombinant protease nexin I. J Biol Chem 266: 22307–22312. 1939253

[65] Blanton RE, Licate LS, Aman RA (1994) Characterization of a native and recombinant *Schistosoma haematobium* serine protease inhibitor gene product. Mol Biochem Parasitol 63: 1–11. 8183308 10.1016/0166-6851(94)90003-5

[66] Mann KG, Brummel-Ziedins K, Orfeo T, Butenas S (2006) Models of blood coagulation. Blood Cells Mol Dis 36: 108–117. 16500122 10.1016/j.bcmd.2005.12.034

[67] Miles LA, Greengard JS, Griffin JH (1983) A comparison of the abilities of plasma kallikrein, beta-factor XIIa, factor XIa and urokinase to activate plasminogen. Thromb Res 29: 407–417. 6344314 10.1016/0049-3848(83)90244-x

[68] Ramajo-Hernandez A, Perez-Sanchez R, Ramajo-Martin V, Oleaga A (2007) *Schistosoma bovis*: Plasminogen binding in adults and the identification of plasminogen-binding proteins from the worm tegument. Exp Parasitol 115: 83–91. 16962583 10.1016/j.exppara.2006.07.003

[69] de la Torre-Escudero E, Manzano-Roman R, Perez-Sanchez R, Siles-Lucas M, Oleaga A (2010) Cloning and characterization of a plasminogen-binding surface-associated enolase from schistosoma bovis. Vet Parasitol 173: 76–84. 20609522 10.1016/j.vetpar.2010.06.011

[70] de la Torre-Escudero E, Manzano-Roman R, Siles-Lucas M, Perez-Sanchez R, Moyano JC, et al (2012) Molecular and functional characterization of a *Schistosoma bovis* annexin: Fibrinolytic and anticoagulant activity. Vet Parasitol 184: 25–36. 21889851 10.1016/j.vetpar.2011.08.013

[71] Skelly PJ, Alan Wilson R (2006) Making sense of the schistosome surface. Adv Parasitol 63: 185–284. 17134654 10.1016/S0065-308X(06)63003-0

[72] Yang J, Qiu C, Xia Y, Yao L, Fu Z, et al (2010) Molecular cloning and functional characterization of *Schistosoma japonicum* enolase which is highly expressed at the schistosomulum stage. Parasitol Res 107: 667–677. 20512506 10.1007/s00436-010-1913-z

[73] Angles-Cano E (1994) Overview on fibrinolysis: Plasminogen activation pathways on fibrin and cell surfaces. Chem Phys Lipids 67–68: 353–362. 10.1016/0009-3084(94)90157-0 8187235

[74] Flood EC, Hajjar KA (2011) The annexin A2 system and vascular homeostasis. Vascul Pharmacol 54: 59–67. 21440088 10.1016/j.vph.2011.03.003 PMC3109204

[75] Pellegrino J, Coelho PM (1978) *Schistosoma mansoni*: Wandering capacity of a worm couple. J Parasitol 64: 181–182. 627964

[76] Salafsky B, Fusco AC (1987) *Schistosoma mansoni*: A comparison of secreted vs nonsecreted eicosanoids in developing schistosomulae and adults. Exp Parasitol 64: 361–367. 2824233 10.1016/0014-4894(87)90048-8

[77] Angeli V, Faveeuw C, Roye O, Fontaine J, Teissier E, et al (2001) Role of the parasite-derived prostaglandin D2 in the inhibition of epidermal langerhans cell migration during schistosomiasis infection. J Exp Med 193: 1135–1147. 11369785 10.1084/jem.193.10.1135 PMC2193325

